# Fc Gamma Receptor IIb on GM-CSF Macrophages Controls Immune Complex Mediated Inhibition of Inflammatory Signals

**DOI:** 10.1371/journal.pone.0110966

**Published:** 2014-10-23

**Authors:** Kim C. M. Santegoets, Mark H. Wenink, Wim B. van den Berg, Timothy R. D. J. Radstake

**Affiliations:** 1 Department of Rheumatology & Clinical Immunology, University Medical Center Utrecht, Utrecht, the Netherlands; 2 Laboratory of Translational Immunology, Department of Immunology, University Medical Center Utrecht, Utrecht, the Netherlands; 3 Department of Rheumatology, Radboud university medical center, Nijmegen, the Netherlands; Institut National de la Santé et de la Recherche Médicale U 872, France

## Abstract

**Background:**

In rheumatoid arthritis (RA) macrophages play a major role in amplifying synovial inflammation. Important activating signals are those induced by Toll-like receptor (TLR) ligands and by activated T cells. The balance between activating and inhibitory Fc gamma receptors (FcγRs) on macrophages might be crucial in modulating these inflammatory responses. The purpose of this study was to determine FcγR expression on pro- and anti-inflammatory macrophages (gmMφ and mMφ, respectively) and identify functional consequences on immune complex uptake and macrophage activation.

**Methods:**

Human monocytes were isolated and differentiated into gmMφ and mMφ. A full FcγR characterization of both macrophage subtypes was performed and uptake of fluorescent immune complexes (ICs) was determined. FcγRIIb isoforms were determined by qPCR. Macrophages were stimulated via different TLRs or cytokine activated T cells in the presence or absence of ICs and cytokine production was determined. Blocking studies were performed to look into the pathways involved.

**Results:**

mMφ expressed high levels of the activating FcγRIIa and FcγRIII and low levels of the inhibitory FcγRIIb, while the FcγR balance on gmMφ was shifted towards the inhibitory FcγRIIb. This was accompanied by a clear increase in FcγRIIb1 mRNA expression in gmMφ. This resulted in higher IC uptake by mMφ compared to gmMφ. Furthermore, FcγR-mediated stimulation of gmMφ inhibited TLR2, 3, 4 and 7/8 mediated cytokine production via FcγRIIb and PI3K signaling. In addition, gmMφ but not mMφ produced TNFα upon co-culture with cytokine activated T cells, which was reduced by IC binding to FcγRIIb. The latter was dependent on PI3K signaling and COX2.

**Conclusions:**

FcγR expression patterns on gmMφ and mMφ are significantly different, which translates in clear functional differences further substantiating FcγRIIb as an interesting target for inflammation control in RA and other autoimmune/inflammatory diseases.

## Introduction

One of the major pathways underlying the pathogenesis of rheumatoid arthritis (RA) is the aberrant production of inflammatory cytokines by macrophages. In the arthritic joint, macrophages are one of the main effector cells present and their levels correlate with disease activity and joint destruction [1], [2]. Their levels are mainly associated with inflammatory cytokines such as TNFα and interleukin (IL) 1β, and could be sustained by factors like granulocyte-macrophage colony-stimulating factor (GM-CSF), present in the RA synovial joint [3]–[5]. Multiple pathways are proposed to play a role in macrophage activation in RA. One mechanism inducing cytokine production by RA macrophages is the triggering of Toll-like receptors (TLRs). Many endogenous TLR ligands have been found in an arthritic joint, such as GP96 and SNAPIN, which activate cells via TLR2, small heat shock protein B8 that can activate TLR4, and self-RNA from damaged cells which is likely to stimulate macrophages via TLR3 or TLR7/8 [6]–[10]. Blocking antibodies against these TLRs reduce spontaneous cytokine production by RA synovial tissue cultures, confirming they are not only present in the arthritic joint but also contribute to the abundant cytokine production seen in RA [10]–[12].

Another pathway mediating synovial macrophage activation is by direct interaction with activated T cells. Cytokine activated T cells resemble RA synovial T cells in their contact-dependent effector function and activation phenotype [13], [14]. These cells can be cultured from peripheral blood lymphocytes in the presence of IL-2, IL-6 and TNFα (cytokine activated T cells, Tck) and induce an unbalanced, inflammatory cytokine response from monocytes [14].

Another component present in many RA patients are auto-antibodies. These can form immune complexes (IC) and especially when deposited in tissues they can activate macrophages. Soluble ICs can have cell activating but also inhibitory effects, as is emphasized by IVIg treatment [15]. An important deciding factor for the cellular response to ICs is the balance of activating and inhibitory Fc gamma receptors (FcγRs).

The FcγR system consists of the activating FcγRI, FcγRIIa and FcγRIII that trigger cell activation via an immunoreceptor tyrosine-based activation motif (ITAM) in their cytoplasmic domain and the inhibitory FcγRIIb that signals via an immunoreceptor tyrosine-based inhibition motif (ITIM) [16]. As the only inhibitory FcγR, FcγRIIb is an important brake on the immune system by inhibition of cell activation via the activating FcγRs on a wide array of cells and inhibition of the B cell receptor. FcγRIIb has two major isoforms, namely FcγRIIb1 and FcγRIIb2, which differ in their capabilities to mediate endocytosis and in their distribution on immune cells [17]–[20]. FcγRIIb1 predominates in B cells, while FcγRIIb2 is the major isoform in myeloid cells. We and others have previously shown that IC binding to FcγRIIb can also inhibit TLR4 signaling [21], [22]. In our previous report, only RA patients that could control their disease activity without the need of anti-rheumatic drugs had high FcγRIIb levels on their dendritic cells (DC) and were capable of this inhibition [21]. This supports an important regulatory role for FcγRIIb in controlling inflammation in RA.

Since proinflammatory macrophages are important in the pathogenic process in RA and there is no data on the expression and function of the inhibitory FcγR on such macrophages we aimed to delineate the expression of FcγR receptors on homeostatic M-CSF macrophages (mMφ) and inflammatory GM-CSF macrophages (gmMφ). We determined the complete FcγR balance on gmMφ and mMφ and tested whether functional differences were attributed to this. We mainly focused on combined FcγR triggering with macrophage activation via a range of TLRs implicated in RA pathology or activated T cells and found that FcγRIIb was able to dampen both TLR and Tck induced TNFα production when this inhibitory FcγRIIb was highly expressed.

## Materials and Methods

### Ethics statement

The study protocol was approved by the medical ethical committee of the Radboud university medical center (Nijmegen, the Netherlands) and the University Medical Center Utrecht (Utrecht, the Netherlands) and all healthy volunteers gave their written informed consent. All experiments were performed in accordance with the Helsinki Declaration.

### Culture of monocyte-derived gmMφ and mMφ and Tck cells

Peripheral blood mononuclear cells were isolated from venous blood of healthy volunteers using density-gradient centrifugation over Ficoll (GE Healthcare, Uppsala, Sweden). Monocytes and CD4+ T cells were obtained using CD14 and CD4 microbeads (Miltenyi Biotec, Bergisch Gladbach, Germany). gmMφ and mMφ were generated by culturing monocytes in the presence of GM-CSF (800 U/ml; R&D Minneapolis, Minnesota, USA) or macrophage colony-stimulating factor (M-CSF, 25 ng/ml; R&D) for 6 days. Macrophages were cultured in 6 well plates (Corning, New York, USA) with 1,0×10^6^ cells per well in 2 ml medium (RPMI-1640 Dutch modification (Gibco Life Technologies, Grand Island, New York, USA)) supplemented with 10% FCS, antibiotic-antimycotic and L-glutamine (Gibco Life Technologies). Culture medium with the same supplements (1 ml) was added at day 3 and the cells were harvested at day 6. In parallel, autologous CD4+ T cells were cultured in complete medium with recombinant human IL-2 (25 ng/ml), IL-6 (100 ng/ml) and TNFα (25 ng/ml) at 2×10^6^ cells/ml for 6 days (all from R&D).

### Phenotypical analysis

Using standardized flow cytometry protocols as described previously gmMφ and mMφ were phenotyped using antibodies against CD14, CD163 (both BD Biosciences, Franklin Lakes, New Jersey, USA) and MHC-II DR/DP (clone Q1514) [23]. FcγR expression was determined with antibodies against FcγRI (CD64, PE labeled, clone 10.1; Dako, Glostrup, Denmark), FcγRIII (CD16, PE labeled, clone DJ130c; Dako), clone IV.3 which preferentially binds to FcγRIIa (StemCell Technologies, Vancouver, Canada) and the FcγRIIb specific antibody 2B6 (Alexa488 labeled; MacroGenics, Rockville, Maryland, USA). Expression of unlabeled markers was visualized via a FITC labeled goat-anti-mouse secondary antibody. Cell fluorescence was measured on a FACS Calibur (BD) and analyzed using Flowjo software for the mean fluorescence intensity (MFI) and the proportion of positive cells relative to cells stained with the appropriate IgG isotypes.

### RNA isolation and qPCR

Total RNA was extracted in 0.5 ml of TRI-reagent and treated with DNase to remove genomic DNA before being reverse-transcribed into cDNA. qPCR was performed on a Quantstudio 12K Flex (Life Technologies) with SYBR Select Master Mix (Life Technologies), 7.5 ng cDNA and a primer concentration of 0.5 µM in a total volume of 15 µl. qPCR signals were quantified by comparing the cycle threshold value (Ct) of the gene of interest of each sample with the Ct value of the reference gene GAPDH (ΔCt). Results were deployed as relative expression (2^−ΔCt^). The following primers were used: GAPDH forward ATGGGGAAGGTGAAGGTCG, reverse GGGGTCATTGATGGCAACAATA; FcγRIIb1 forward GGATTTCAGCTCTCCCAGGAT, reverse CGGTTCTGGTCATCAGGCTC; FcγRIIb2 forward AAAGCGGATTTCAGCCAATC, reverse CAAGACAATGGAGACTAAATACGGT.

### Phagocytosis and binding assay

Phagocytosis assays were performed with fluorescently labeled ICs, prepared as previously described [24]. Macrophages were incubated with FITC-labeled ICs (50 µg/ml) for 30 min at 4°C and 37°C to determine binding and uptake, respectively. Unattached ICs were washed away before determining binding and uptake by flow cytometry. To determine IC uptake extracellular attached FITC-IC was quenched by adding trypan blue (1/40 diluted in PBS, Sigma-Aldrich) to the samples just before determining the IC uptake by flow cytometry.

### Stimulation of monocyte-derived macrophages

At day 6 macrophages were harvested and plated in a concentration of 0.5×10^6^ cells/ml in 96 well culture plates (100 µl). Immune complexes used in this study were prepared by heating human IgG (Sigma-Aldrich, St. Louis, Missouri, USA) in PBS at 63°C for 30 minutes (heat-aggregated immune complexes (IC)), as previously described [25] and were used in a concentration of 50 µg/ml. Macrophages were stimulated or not with ICs for 15–30 minutes before the addition of TLR agonists for 20 hours. The following concentrations of TLR agonists were used: Pam3CSK4 (5 µg/ml, EMC Microcollections, Tübingen, Germany), Poly(I:C) (25 µg/ml, Invivogen, San Diego, California, USA), LPS (100 ng/ml, E. coli 0111:B4, Sigma-Aldrich) and R848 (2 µg/ml, Invivogen) for TLR2/1, 3, 4 and 7/8 respectively. The LPS was double-purified to remove any contaminating proteins as described previously [26]. Macrophages were also co-cultured with cytokine-activated T cells for 20 hours in a 1∶5 ratio in the presence or absence of IC (50 µg/ml) prestimulation for 1 h.

FcγRIIb blocking was performed by 30 min incubation of mφ-1 with 10 µg/ml 2B6 antibody (MacroGenics) or an isotype control at 4°C before stimulation with ICs and LPS or Tck. In other experiments gmMφ were treated with the PI3K inhibitors Wortmannin (0.1 µM; Calbiochem, San Diego, California, USA) or LY294002 (10 µM; Calbiochem) or COX2 inhibitor I (20 µM; Calbiochem) for 1 h at 37°C before stimulation.

### Measurement of cytokines in culture supernatants

Levels of IL-10 and TNFα were measured in the supernatants using commercially available kits (Millipore, Billerica, Massachusetts, USA) according to the manufacturer’s instructions. Cytokine levels were measured and analyzed with the Bio-Plex system (Bio-Rad, Hercules, California, USA).

### Statistical analysis

Differences were analyzed using paired Student’s t-tests. P values less than 0.05 were considered significant.

## Results

### gmMφ express high levels of the inhibitory FcγRIIb, while mMφ express higher levels of the activating FcγRIIa and FcγRIII

**Figure 1:**
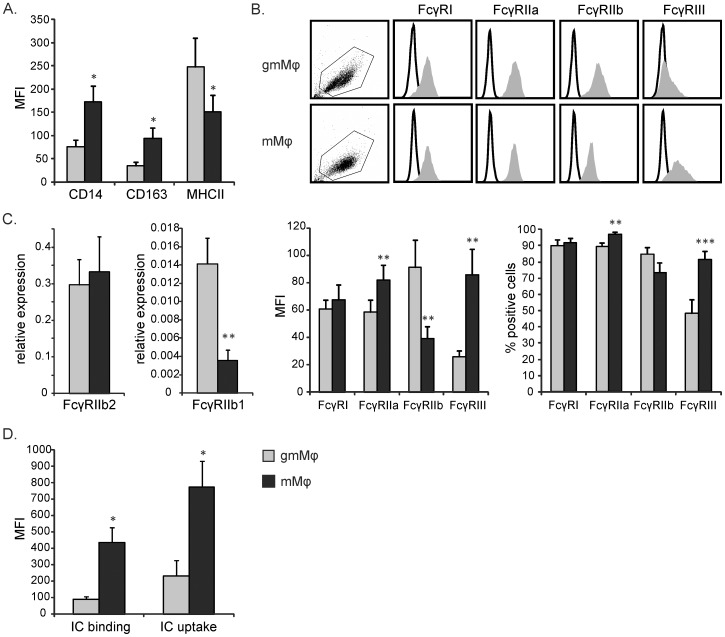
gmMφ express high FcγRIIb levels, while mMφ express more FcγRIIa and FcγRIII. Monocytes were cultured for 6 days with GM-CSF or M-CSF into gmMφ and mMφ respectively. Expression of CD14, CD163 and MHC-II (**A**) and all FcγRs (**B**) was determined by flow cytometry. (**B**) For FcγR expression, representative FACS plots are shown together with bar graphs showing mean (and SEM of) MFI and percentage of positive cells from 11 donors. Histograms show isotype control (thin line) and FcγR specific antibody (solid grey). (**C**) mRNA expression of FcγRIIb1 and FcγRIIb2 were determined by qPCR and plotted as relative expression compared to GAPDH. Bars are mean and SEM of 7 donors. (**D**) gmMφ and mMφ were incubated with FITC-labeled ICs (50 µg/ml) for 30 min at 4°C for binding and 37°C for uptake. IC uptake was determined in the presence of trypan blue. Bars are mean and SEM from 4 donors. *P<0.05, **P<0.01 and ***P<0.001 compared to mφ-1.

Monocytes were cultured into pro- and anti-inflammatory gmMφ and mMφ in the presence of either GM-CSF or M-CSF. To confirm the phenotype of our gmMφ and mMφ we first analyzed their expression of CD14, CD163 and MHC-II. In line with literature the expression of CD14 and CD163 was higher on mMφ, while MHC-II was increased on gmMφ (Figure 1A) [21]. We further evaluated the expression of activating and inhibitory FcγRs. The monomeric IgG receptor FcγRI was similarly expressed in gmMφ and mMφ, while FcγRIII expression was highly increased on mMφ compared to gmMφ (regarding both MFI and percentage of positive cells, Figure 1B). Investigating the activating and inhibiting subtype of FcγRII separately, we observed a marked difference between the gmMφ en mMφ. Whereas the activating FcγRIIa is expressed higher on mMφ, expression of the inhibitory FcγRIIb was increased on gmMφ (Figure 1B). More specifically, the FcγRIIb/FcγRIIa ratio was 1.56 for gmMφ and 0.48 for mMφ. Thus, gmMφ display an FcγR balance favored towards the inhibitory subtype whereas the opposite was found on mMφ. FcγR expression was also determined on gmMφ and mMφ from some RA patients, which showed a similar FcγR distribution compared to healthy controls (data not shown). In vivo in situations in which GM-CSF is produced most likely also a basal level of M-CSF will be present. To determine the effect of the combination of both growth factors on macrophage development we also cultured macrophages with GM-CSF and M-CSF. This resulted in a phenotype similar to gmMφ, suggesting GM-CSF is dominant over M-CSF, at least regarding FcγR expression (data not shown).

We further aimed to differentiate between the two major FcγRIIb isoforms, FcγRIIb1 and FcγRIIb2. Since the extracellular domain of these isoforms is the same we used qPCR to determine the expression of these variants in mMφ and gmMφ. Using isoform specific primers we found that FcγRIIb2 expression was similar in both macrophage subtypes, while FcγRIIb1 expression was significantly increased in gmMφ compared to mMφ (Figure 1C). gmMφ thus have an increased expression of the FcγRIIb variant usually more predominant in B cells which is less capable of mediating endocytosis.

The capacity to take up ICs is an important function of macrophages. To evaluate the functionality of the altered aforementioned FcγR balance, we investigated whether the gmMφ and mMφ display a different binding and uptake capacity of ICs. mMφ show a significantly increased potential for both binding and uptake of ICs compared to gmMφ (Figure 1D). This is fitting with the enhanced expression of FcγRIIa and FcγRIII on mMφ, which have a higher affinity for most IgG isotypes compared to FcγRIIb [27] and the increased expression of the non-endocytosing FcγRIIb1 on gmMφ.

### ICs inhibit TLR induced cytokine production by gmMφ but not by mMφ

**Figure 2:**
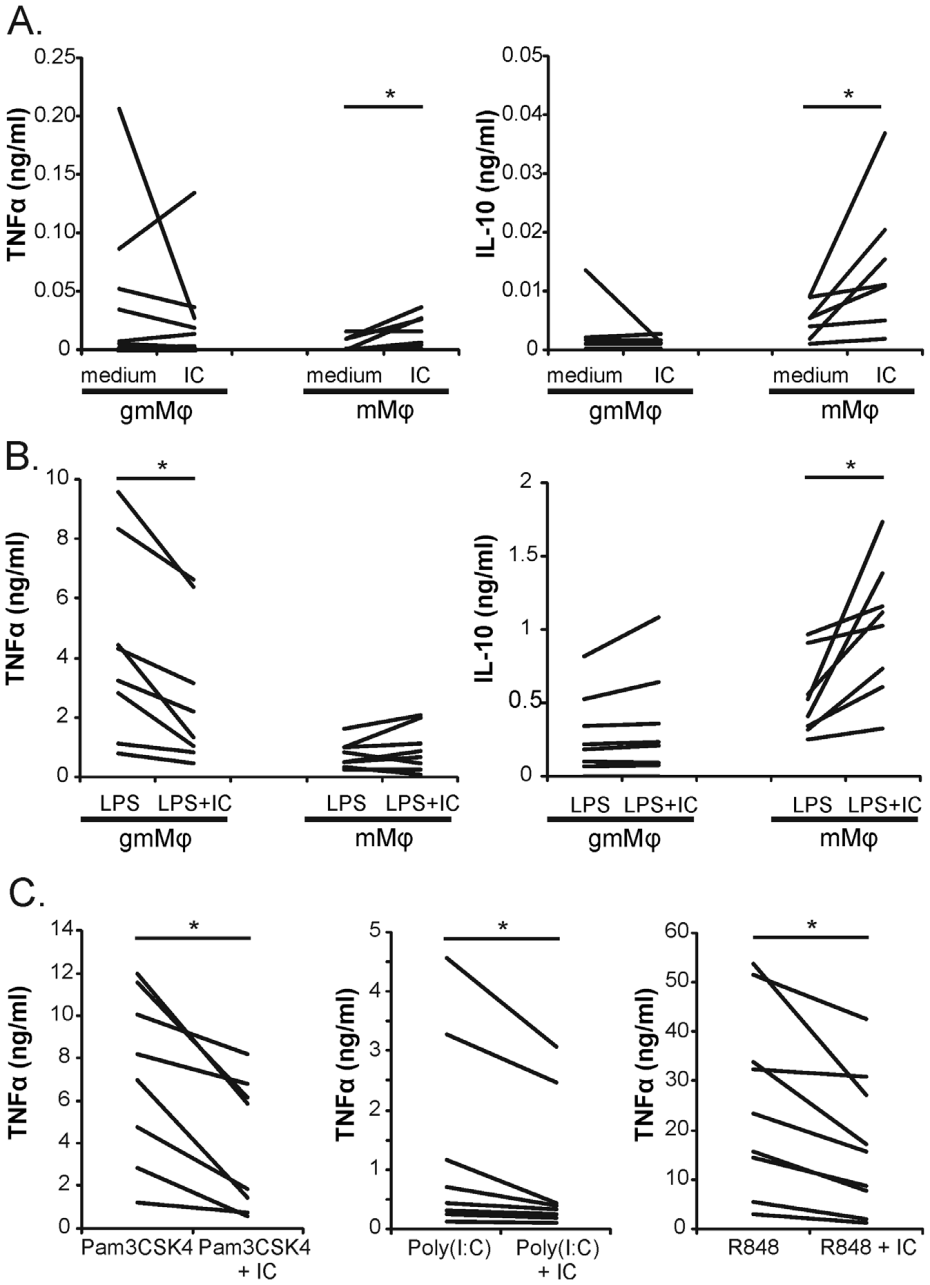
Immune complexes can inhibit TLR2, 3, 4 and 7/8 induced cytokine production in gmMφ. gmMφ and mMφ were stimulated with ICs (50 µg/ml) (**A**), LPS (100 ng/ml) or LPS+ICs (**B**) and TNFα and IL-10 were measured in culture supernatants after 20 hours. (**C**) gmMφ were stimulated with Pam3CSK4 (5 µg/ml), Poly(I:C) (25 µg/ml) or R848 (2 µg/ml) in the presence or absence of ICs. After 20 hours, supernatants were collected and analyzed for TNFα levels. Figure shows data of at least 7 donors for all stimulations. *P<0.05 difference with and without IC.

To further evaluate the functional consequences of the differential FcγR expression on gmMφ and mMφ and to test whether ICs can also inhibit TLR4 signaling in human macrophages that express high FcγRIIb levels, gmMφ and mMφ were stimulated with TLR ligands in combination with ICs. mMφ and gmMφ were first stimulated with ICs alone to determine the effect of differential FcγR expression on these cells on IC induced cytokine production. In mMφ ICs induced significant but low levels of TNFα and IL-10 production, while there was no clear cytokine induction observed in gmMφ (Figure 2A). Upon TLR 4 stimulation with LPS gmMφ produced high levels of TNFα and low levels of IL-10, while mMφ were marked by their relatively high IL-10 production and low production of TNFα which corroborates the literature (Figure 2B) [5], [28]. After co-stimulation with ICs, gmMφ were able to significantly attenuate TNFα production compared to those stimulated with LPS alone, while IL-10 production was relatively unaffected (Figure 2B). In contrast, but in line with our observations on FcγR expression, the addition of ICs to LPS did not result in inhibition of TLR4 mediated cytokine production in mMφ. In fact, mMφ produced significantly more IL-10 after co-stimulation with ICs.

To determine if this inhibitory pathway can also affect cytokine induction by other TLR ligands, similar experiments were performed with specific ligands for TLR2/1 (Pam3CSK4), TLR3 (Poly(I:C)) and TLR7/8 (R848). These experiments learned that the inhibitory effect of ICs on TLR signaling by gmMφ is not limited to TLR4, but also extends to TLR2/1, TLR3 and TLR7/8 (Figure 2C), further substantiating the pivotal role of the FcγR balance in the regulation of cell activation. Again, IL-10 production by gmMφ was not clearly affected by the presence of ICs and the inhibitory effect on TNFα production was not present in mMφ (data not shown). To determine if ICs could also affect TLR induced cytokine production after TLR stimulation has already occurred, we performed a time course with addition of ICs from 2 hours prior to Pam3CSK4 till 2 hours after Pam3CSK stimulation. ICs were able to significantly modulate TNFα production during the whole time range, without significantly affecting IL-10 (data not shown). ICs can thus modulate TLR induced cytokine production before and after TLR triggering.

### Immune complexes can inhibit gmMφ activation by activated T cells

**Figure 3:**
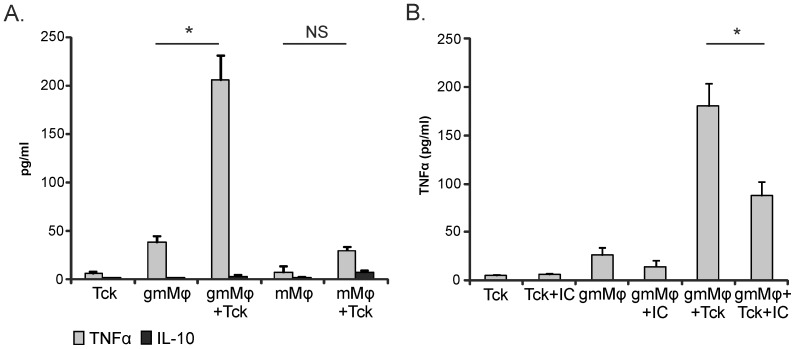
IC can inhibit T cell mediated macrophage activation in gmMφ. gmMφ or mMφ and Tck were cultured from the same donor. (**A**) At day 6 the macrophages and Tcks were harvested, washed and cultured together in a ratio of 1∶5. TNFα and IL-10 were measured in the supernatant after 20 hours. Bars are mean and SEM from 3 independent experiments. (**B**) gmMφ were cultured alone or in a 1∶5 ratio with Tck in the presence or absence of ICs (50 µg/ml) for 20 hours before collecting the supernatant. Bars are mean and SEM from 6 independent experiments. *P<0.05, NS is not significant.

Another important activator of RA synovial macrophages are cytokine activated T cells. We therefore evaluated cytokine production in co-cultures of Tck with gmMφ or mMφ. Tck induced a synergistic production of TNFα when co-cultured with gmMφ (Figure 3A), while IL-10 is almost absent, resulting in an unbalanced proinflammatory response. The TNFα production by mMφ after co-culture with Tck is much lower and not significantly different from mMφ alone (Figure 3A). In contrast to mMφ stimulation by TLR ligands, in co-culture with Tck also IL-10 production remained low. Thus, Tck mainly stimulate gmMφ. To determine if ICs could inhibit Tck induced TNFα production when macrophages express high FcγRIIb levels, gmMφ were stimulated with ICs and Tck. IC co-stimulation reduced the TNFα release by gmMφ with approximately 50% upon Tck stimulation (Figure 3B). ICs can thus modulate both TLR and T cell induced gmMφ activation.

### The inhibitory effect of ICs is mediated via FcγRIIb and the PI3K pathway

**Figure 4:**
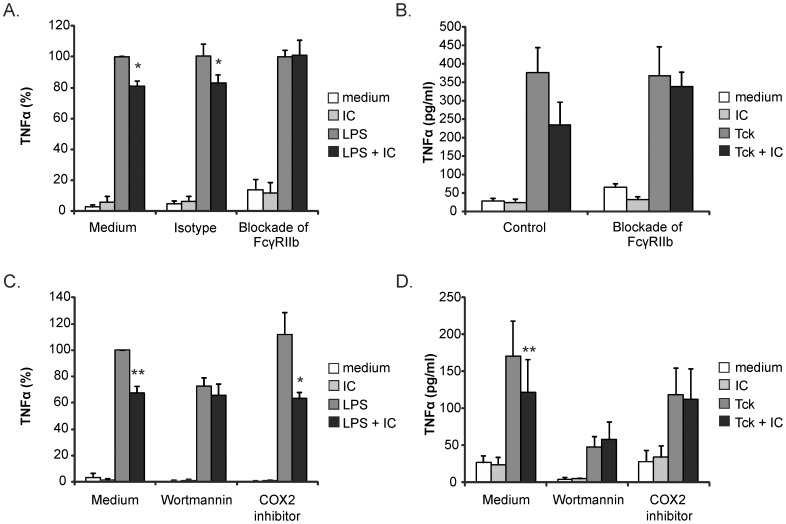
TLR and Tck inhibition by IC is mediated via FcγRIIb and PI3K. gmMφ were pre-incubated for 30 minutes with the FcγRIIb specific blocking antibody 2B6 (10 µg/ml) or an isotype control before stimulation with IC (50 µg/ml) and LPS (100 ng/ml) (**A**) or Tck (**B**). gmMφ were pre-incubated with Wortmannin (0.1 µM) or a Cox2 inhibitor (20 µM) for 1 hr before stimulation with IC and LPS (**C**) or Tck (**D**). 20 hour supernatants were collected to analyze TNFα levels. In the graphs showing TLR stimulation (A and C) the percentage of TNFα production is plotted with the LPS only stimulation set at 100%. In the Tck graphs (B and D) absolute values are shown. All graphs show the mean and SEM of at least 3 experiments. *P<0.05.

To confirm whether the high FcγRIIb expression on gmMφ was indeed responsible for the inhibitory effect of ICs on TLR and Tck induced signaling in these cells, we used a blocking antibody against FcγRIIb. Blocking of FcγRIIb fully abrogated the inhibitory effect of ICs on both TLR and Tck induced TNFα production (Figure 4A and B).

In DCs our group has previously shown that the PI3K/Akt pathway is involved in the crosstalk between FcγRIIb and TLR4 [21]. To determine if this pathway is also involved in the FcγRIIb effect on macrophages, we blocked PI3K signaling before stimulation of gmMφ with ICs and LPS. The IC mediated inhibition of LPS induced TNFα production was abrogated in the presence of Wortmannin or LY294002, confirming the role of the PI3K pathway in FcγRIIb mediated TLR4 signaling inhibition in gmMφ (Figure 4C and data not shown). Mice studies pointed towards an additional role for prostaglandin E2 in the inhibitory actions of FcγRIIb on TLR4 signaling [22]. To test this in the human setting we performed our experiments in the presence of a COX2 inhibitor. This did not affect the IC mediated inhibition of cytokine production by gmMφ upon TLR4 stimulation (Figure 4C). To test if the same mechanism was involved in IC mediated blocking of other TLRs we tested FcγRIIb blocking and PI3K inhibition also for ICs in combination with TLR2/1 stimulation and this gave similar results as shown for TLR4 (data not shown).

To further determine if similar pathways are involved in inhibition of Tck induced macrophage activation we performed the Tck experiments in the presence of Wortmannin or a COX2 inhibitor. As for TLR activation a functioning PI3K pathway was necessary for the inhibitory effect of ICs, however IC mediated blocking of gmMφ activation via Tck also appeared to be dependent on prostaglandin production, as is exemplified by the lack of IC mediated inhibition in the presence of a COX2 inhibitor (Figure 4D). IC mediated inhibition of gmMφ TNFα production via both pathways is mediated via binding to FcγRIIb and involves the PI3K pathway. Prostaglandins are necessary for the effect of IC when combined with Tck, but not for TLR mediated cell activation.

## Discussion

The present study shows that gmMφ have a relatively high expression of FcγRIIb compared to the activating FcγRs, while this balance is shifted towards the activating FcγRs on mMφ. gmMφ secrete large amounts of TNFα upon stimulations relevant in RA, such as TLR ligands and cytokine activated T cells. Under these conditions inhibitory immune receptors, such as FcγRIIb, are crucial to counter-regulate the induced inflammatory responses to prevent excessive tissue damage. We show that the switched balance towards the inhibitory FcγRIIb on gmMφ is functionally relevant and can inhibit TNFα secretion from these cells induced by either TLRs or Tck in the presence of soluble ICs. This way it could function as a natural brake in an attempt to prevent excessive cytokine production and inflammation in RA.

The important regulatory role of FcγRIIb is extensively shown in animal models for autoimmunity (Reviewed in [29]). In this context it was shown that the transfer of RA but not healthy control serum can induce arthritis in FcγRIIb^−/−^, but not in normal B6 mice [30]. This was caused by the IgG portion supporting a pathogenic role for IgG (auto) antibodies from RA patients and an important regulatory role for FcγRIIb. This model bypasses the effect of B cells because human IgG is passively transferred and it thus shows that FcγRIIb expression on other effector cells, including macrophages and DCs, is crucial to prevent autoimmune inflammation.

We demonstrated for the first time that FcγRIIb can inhibit cytokine induction by a wide range of TLRs, of which ligands have been found in the arthritic joint, including TLR2, TLR3, TLR4 and TLR7/8. In addition, FcγRIIb can also inhibit macrophage TNFα production induced by activated T cells. This way FcγRIIb can actively control two important stimulatory pathways for macrophages in RA. Inhibitor studies taught us that normal PI3K signaling is necessary for FcγRIIb inhibition of both TLR and Tck induced cytokine release, while prostaglandins are only involved in the latter. Prostaglandins were postulated as an essential signaling molecule in FcγRIIb mediated inhibition of TLR4 in mouse macrophages [22]. However, in our human experimental setting prostaglandins are dispensable for FcγRIIb mediated inhibition of TLRs. In our cultures prestimulation of gmMφ with ICs for only 15–30 minutes or up to 2 hours after TLR stimulation was enough to get inhibition, while in mouse macrophages the dependency on prostaglandins was demonstrated after 24 hour prestimulation with ICs [22]. So prostaglandins are not necessary for the direct inhibition of TLR4 signaling by FcγRIIb in humans, but might have additional inhibitory effects at later time points. This would be in line with the dependency of Tck inhibition on prostaglandin production, since the induction of TNFα in this setting is described to be much slower (peaks at 24 hrs.) compared to TLR stimulated TNFα induction (peaks at 4–8 hrs.) [31]. However, much is still unknown about the pathways involved in macrophage activation upon interaction with Tcks. CD69, CD18 and CD49d on the Tck were shown to be involved in the induction of TNFα by monocytes upon Tck co-culture [14]. On monocytes/macrophages ICAM-1 and VCAM-1 might be involved as binding partners for CD18 and CD49d, respectively. Thus far, no direct interactions are known between these molecules and FcγR signaling. Hence, our work justifies more research focused at deciphering potential mechanisms involved in FcγRIIb inhibition on T cell mediated macrophage activation.

Interestingly, the increased membrane FcγRIIb expression on gmMφ coincides with an increased expression of FcγRIIb1 on mRNA level. This suggests that FcγRIIb1 expression on gmMφ could play a role in the inhibitory effects of ICs on these cells. Although the FcγRIIb isoforms have been repeatedly shown to have differential endocytosis potential [17]–[19], not much is known about possible differences in inhibitory signaling. It has been described that FcγRIIb1 is differently phosphorylated in B cells compared to FcγRIIb2 and might have additional inhibitory functions [19], [32], but this has not been repeated by another group in macrophage cell lines [33]. Whether this could have functional implications for macrophage responses towards ICs needs to be further investigated.

Some groups have tried to identify the macrophage phenotype or the FcγR expression on macrophages from RA synovial tissue. FcγRII overall, FcγRIIb in particular and FcγRIII were all increased in RA synovium and correlated with the amount of macrophages present [34], [35]. Looking at in vitro markers for gmMφ and mMφ it remains difficult to fully characterize the macrophages from the synovial tissue since they express markers representing both phenotypes [36], [37]. Supported by our data the expression of FcγRIIb could be an additional marker for gmMφ while FcγRIII marks mMφ macrophages. The ratio between these two FcγRs could be a good discriminator between these macrophage subsets. The high expression of activating FcγRs by mMφ, which are mainly described for their controlling/homeostatic functions, and the high expression of the inhibitory FcγRIIb on the more inflammatory gmMφ might seem contradictory. However, because of the easily activated phenotype of gmMφ regulatory mechanisms including those via FcγRIIb are crucial to prevent excessive inflammation and tissue damage. In addition, the capacity of mMφ to remove IC is facilitated by the high expression of FcγRs, preventing accumulation of IC and thereby preventing unwanted inflammatory responses. Next to that we show that small ICs in combination with TLR stimulation mainly increase IL-10 production in mMφ and no major induction of TNFα was observed.

The in vivo situation is not as black and white as shown by this in vitro model, but knowledge about functional characteristics of these macrophage subsets combined with more detailed phenotyping of local macrophages, including differentiation between FcγRIIa and FcγRIIb in different diseases might give clues about the pathogenic processes going on in vivo. This could possibly give leads for therapeutic options to increase FcγRIIb expression on macrophages even further to induce a more inhibitory phenotype.

## Conclusions

gmMφ and mMφ are characterized by a different FcγR balance, with high FcγRIIa and FcγRIII levels on mMφ and increased FcγRIIb expression on pro-inflammatory gmMφ. The relatively high FcγRIIb expression on gmMφ makes these cells sensitive to IC mediated inhibition of proinflammatory cytokine release upon stimulation by TLR ligands and Tck, which can be an important feedback mechanism to prevent excessive inflammation. This shows that FcγRIIb mediated cell inhibition is not restricted to ITAM containing receptors or TLR4, but can broadly regulate immune responses. Specific targeting of FcγRIIb might therefore open novel therapeutic avenues for RA and other chronic immune mediated inflammatory disorders.
